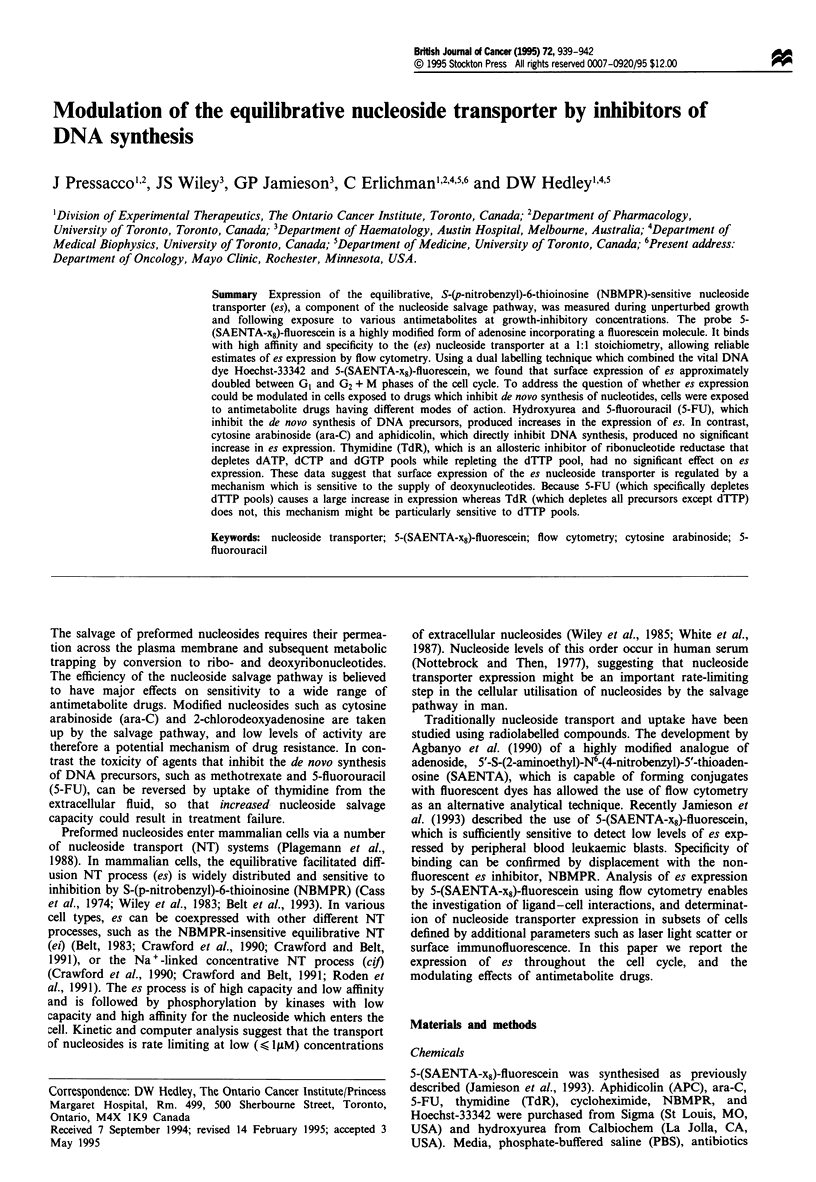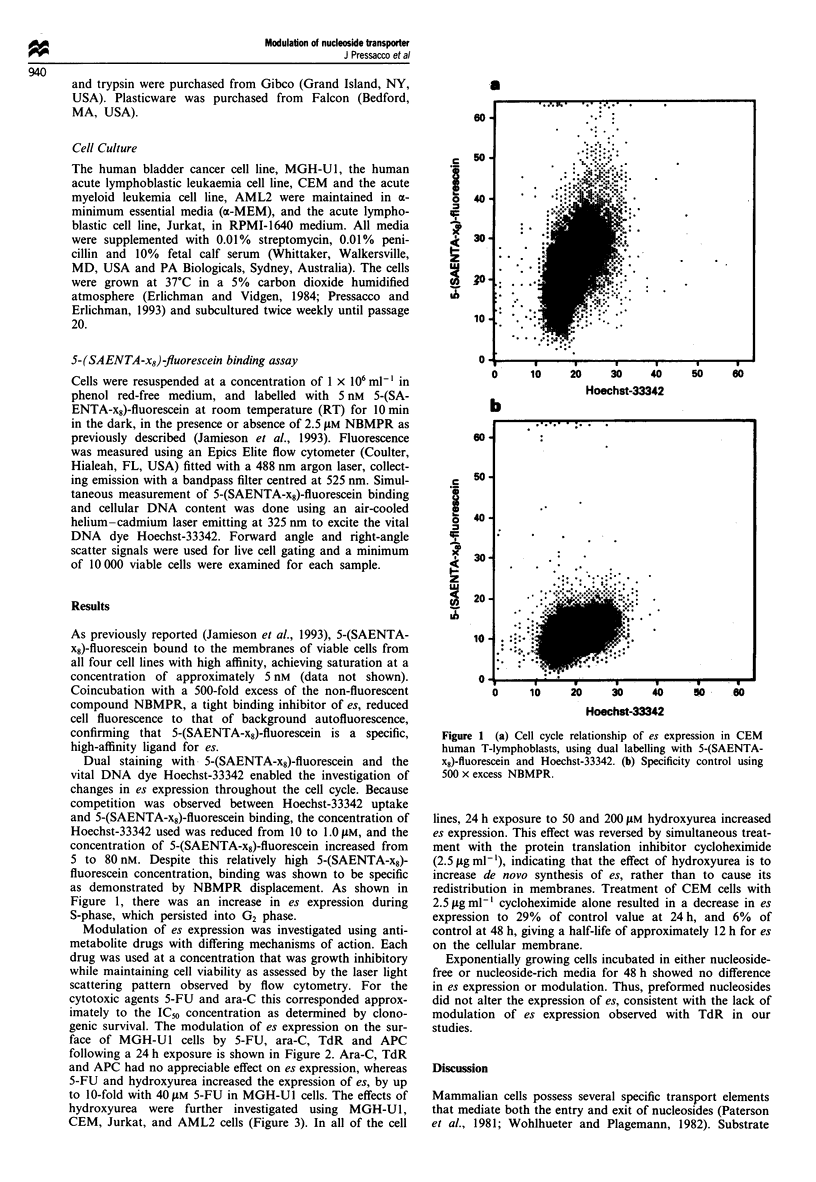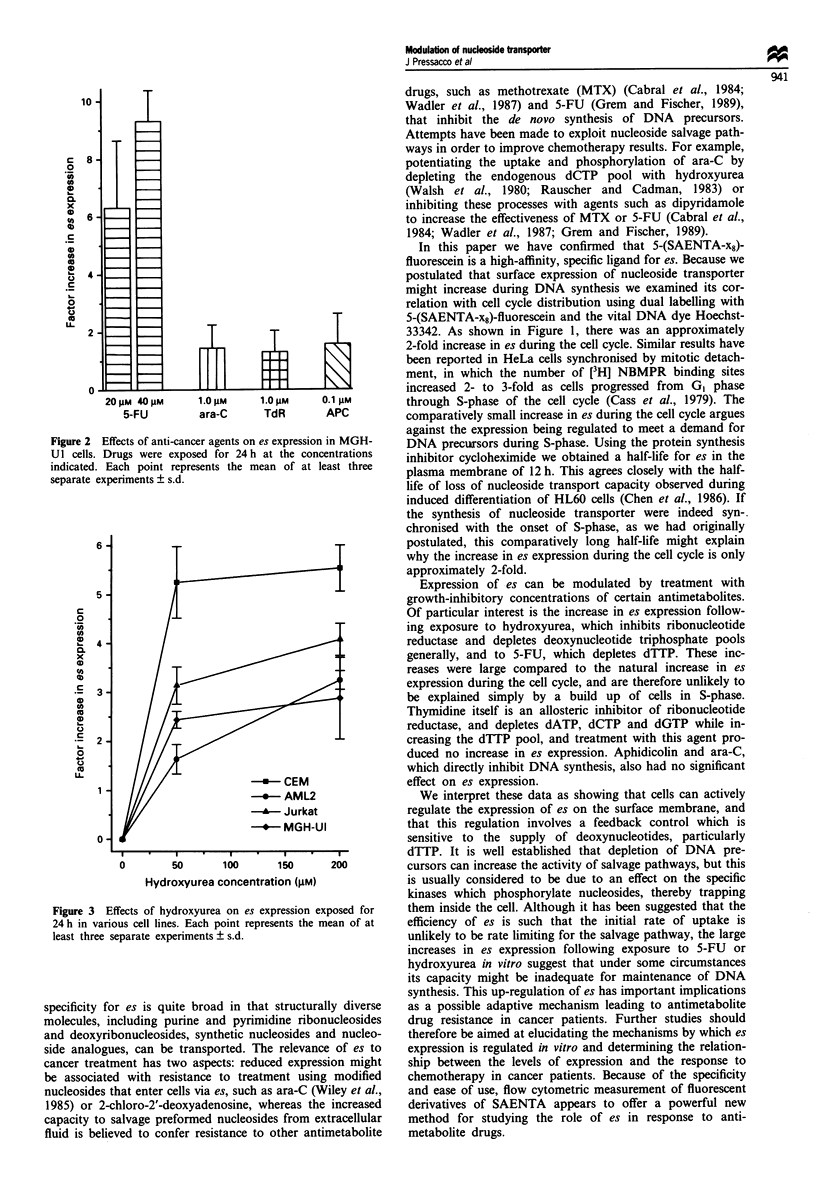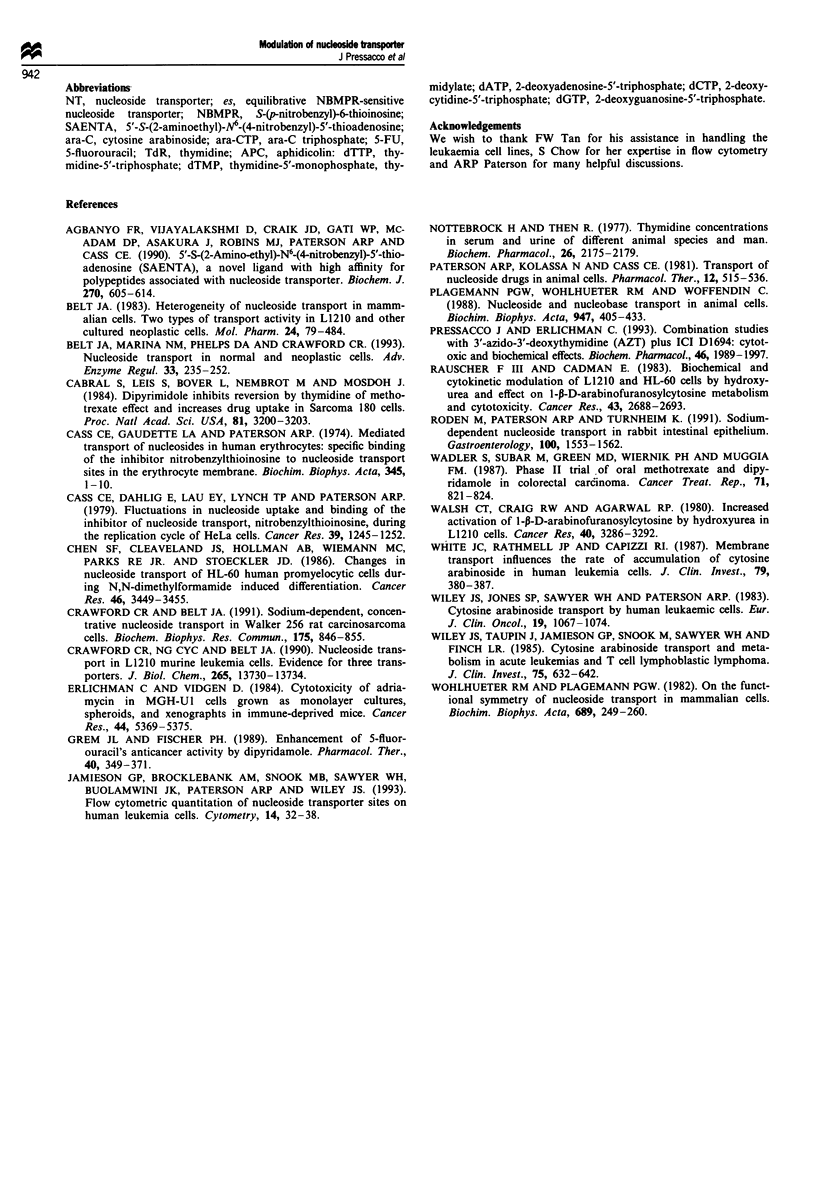# Modulation of the equilibrative nucleoside transporter by inhibitors of DNA synthesis.

**DOI:** 10.1038/bjc.1995.437

**Published:** 1995-10

**Authors:** J. Pressacco, J. S. Wiley, G. P. Jamieson, C. Erlichman, D. W. Hedley

**Affiliations:** Division of Experimental Therapeutics, Ontario Cancer Institute, Toronto, Canada.

## Abstract

Expression of the equilibrative, S-(p-nitrobenzyl)-6-thioinosine (NBMPR)-sensitive nucleoside transporter (es), a component of the nucleoside salvage pathway, was measured during unperturbed growth and following exposure to various antimetabolites at growth-inhibitory concentrations. The probe 5-(SAENTA-x8)-fluorescein is a highly modified form of adenosine incorporating a fluorescein molecule. It binds. with high affinity and specificity to the (es) nucleoside transporter at a 1:1 stoichiometry, allowing reliable estimates of es expression by flow cytometry. Using a dual labelling technique which combined the vital DNA dye Hoechst-33342 and 5-(SAENTA-x8)-fluorescein, we found that surface expression of es approximately doubled between G1 and G2 + M phases of the cell cycle. To address the question of whether es expression could be modulated in cells exposed to drugs which inhibit de novo synthesis of nucleotides, cells were exposed to antimetabolite drugs having different modes of action. Hydroxyurea and 5-fluorouracil (5-FU), which inhibit the de novo synthesis of DNA precursors, produced increases in the expression of es. In contrast, cytosine arabinoside (ara-C) and aphidicolin, which directly inhibit DNA synthesis, produced no significant increase in es expression. Thymidine (TdR), which is an allosteric inhibitor of ribonucleotide reductase that depletes dATP, dCTP and dGTP pools while repleting the dTTP pool, had no significant effect on es expression. These data suggest that surface expression of the es nucleoside transporter is regulated by a mechanism which is sensitive to the supply of deoxynucleotides. Because 5-FU (which specifically depletes dTTP pools) causes a large increase in expression whereas TdR (which depletes all precursors except dTTP) does not, this mechanism might be particularly sensitive to dTTP pools.


British Journal of Cancer (1995) 72, 939–942
© 1995 Stockton Press All rights reserved 0007-0920/95 $12.00

### Modulation of the equilibrative nucleoside transporter by inhibitors of DNA synthesis

J Pressacco[1,2], JS Wiley[3], GP Jamieson[3], C Erlichman[1,2,4,5,6] and DW Hedley[1,4,5]

[1]*Division of Experimental Therapeutics, The Ontario Cancer Institute, Toronto, Canada;* [2]*Department of Pharmacology, University of Toronto, Toronto, Canada;* [3]*Department of Haematology, Austin Hospital, Melbourne, Australia;* [4]*Department of Medical Biophysics, University of Toronto, Canada;* [5]*Department of Medicine, University of Toronto, Canada;* [6]*Present address: Department of Oncology, Mayo Clinic, Rochester, Minnesota, USA.*

**Summary** Expression of the equilibrative, *S*-(*p*-nitrobenzyl)-6-thioinosine (NBMPR)-sensitive nucleoside transporter (*es*), a component of the nucleoside salvage pathway, was measured during unperturbed growth and following exposure to various antimetabolites at growth-inhibitory concentrations. The probe 5-(SAENTA-$x_8$)-fluorescein is a highly modified form of adenosine incorporating a fluorescein molecule. It binds with high affinity and specificity to the (*es*) nucleoside transporter at a 1:1 stoichiometry, allowing reliable estimates of *es* expression by flow cytometry. Using a dual labelling technique which combined the vital DNA dye Hoechst-33342 and 5-(SAENTA-$x_8$)-fluorescein, we found that surface expression of *es* approximately doubled between $G_1$ and $G_2 + M$ phases of the cell cycle. To address the question of whether *es* expression could be modulated in cells exposed to drugs which inhibit *de novo* synthesis of nucleotides, cells were exposed to antimetabolite drugs having different modes of action. Hydroxyurea and 5-fluorouracil (5-FU), which inhibit the *de novo* synthesis of DNA precursors, produced increases in the expression of *es*. In contrast, cytosine arabinoside (ara-C) and aphidicolin, which directly inhibit DNA synthesis, produced no significant increase in *es* expression. Thymidine (TdR), which is an allosteric inhibitor of ribonucleotide reductase that depletes dATP, dCTP and dGTP pools while repleting the dTTP pool, had no significant effect on *es* expression. These data suggest that surface expression of the *es* nucleoside transporter is regulated by a mechanism which is sensitive to the supply of deoxynucleotides. Because 5-FU (which specifically depletes dTTP pools) causes a large increase in expression whereas TdR (which depletes all precursors except dTTP) does not, this mechanism might be particularly sensitive to dTTP pools.

**Keywords:** nucleoside transporter; 5-(SAENTA-$x_8$)-fluorescein; flow cytometry; cytosine arabinoside; 5-fluorouracil

The salvage of preformed nucleosides requires their permeation across the plasma membrane and subsequent metabolic trapping by conversion to ribo- and deoxyribonucleotides. The efficiency of the nucleoside salvage pathway is believed to have major effects on sensitivity to a wide range of antimetabolite drugs. Modified nucleosides such as cytosine arabinoside (ara-C) and 2-chlorodeoxyadenosine are taken up by the salvage pathway, and low levels of activity are therefore a potential mechanism of drug resistance. In contrast the toxicity of agents that inhibit the *de novo* synthesis of DNA precursors, such as methotrexate and 5-fluorouracil (5-FU), can be reversed by uptake of thymidine from the extracellular fluid, so that *increased* nucleoside salvage capacity could result in treatment failure.

Preformed nucleosides enter mammalian cells via a number of nucleoside transport (NT) systems (Plagemann *et al.*, 1988). In mammalian cells, the equilibrative facilitated diffusion NT process (*es*) is widely distributed and sensitive to inhibition by S-(p-nitrobenzyl)-6-thioinosine (NBMPR) (Cass *et al.*, 1974; Wiley *et al.*, 1983; Belt *et al.*, 1993). In various cell types, *es* can be coexpressed with other different NT processes, such as the NBMPR-insensitive equilibrative NT (*ei*) (Belt, 1983; Crawford *et al.*, 1990; Crawford and Belt, 1991), or the $Na^+$-linked concentrative NT process (*cif*) (Crawford *et al.*, 1990; Crawford and Belt, 1991; Roden *et al.*, 1991). The *es* process is of high capacity and low affinity and is followed by phosphorylation by kinases with low capacity and high affinity for the nucleoside which enters the cell. Kinetic and computer analysis suggest that the transport of nucleosides is rate limiting at low ($\leqslant 1 \mu M$) concentrations of extracellular nucleosides (Wiley *et al.*, 1985; White *et al.*, 1987). Nucleoside levels of this order occur in human serum (Nottebrock and Then, 1977), suggesting that nucleoside transporter expression might be an important rate-limiting step in the cellular utilisation of nucleosides by the salvage pathway in man.

Traditionally nucleoside transport and uptake have been studied using radiolabelled compounds. The development by Agbanyo *et al.* (1990) of a highly modified analogue of adenoside, 5′-S-(2-aminoethyl)-$N^6$-(4-nitrobenzyl)-5′-thioadenosine (SAENTA), which is capable of forming conjugates with fluorescent dyes has allowed the use of flow cytometry as an alternative analytical technique. Recently Jamieson *et al.* (1993) described the use of 5-(SAENTA-$x_8$)-fluorescein, which is sufficiently sensitive to detect low levels of *es* expressed by peripheral blood leukaemic blasts. Specificity of binding can be confirmed by displacement with the non-fluorescent *es* inhibitor, NBMPR. Analysis of *es* expression by 5-(SAENTA-$x_8$)-fluorescein using flow cytometry enables the investigation of ligand–cell interactions, and determination of nucleoside transporter expression in subsets of cells defined by additional parameters such as laser light scatter or surface immunofluorescence. In this paper we report the expression of *es* throughout the cell cycle, and the modulating effects of antimetabolite drugs.

### Materials and methods

### *Chemicals*

5-(SAENTA-$x_8$)-fluorescein was synthesised as previously described (Jamieson *et al.*, 1993). Aphidicolin (APC), ara-C, 5-FU, thymidine (TdR), cycloheximide, NBMPR, and Hoechst-33342 were purchased from Sigma (St Louis, MO, USA) and hydroxyurea from Calbiochem (La Jolla, CA, USA). Media, phosphate-buffered saline (PBS), antibiotics

Correspondence: DW Hedley, The Ontario Cancer Institute/Princess Margaret Hospital, Rm. 499, 500 Sherbourne Street, Toronto, Ontario, M4X 1K9 Canada
Received 7 September 1994; revised 14 February 1995; accepted 3 May 1995

and trypsin were purchased from Gibco (Grand Island, NY, USA). Plasticware was purchased from Falcon (Bedford, MA, USA).

*Cell Culture*

The human bladder cancer cell line, MGH-U1, the human acute lymphoblastic leukaemia cell line, CEM and the acute myeloid leukemia cell line, AML2 were maintained in α-minimum essential media (α-MEM), and the acute lymphoblastic cell line, Jurkat, in RPMI-1640 medium. All media were supplemented with 0.01% streptomycin, 0.01% penicillin and 10% fetal calf serum (Whittaker, Walkersville, MD, USA and PA Biologicals, Sydney, Australia). The cells were grown at 37°C in a 5% carbon dioxide humidified atmosphere (Erlichman and Vidgen, 1984; Pressacco and Erlichman, 1993) and subcultured twice weekly until passage 20.

*5-(SAENTA-$x_8$)-fluorescein binding assay*

Cells were resuspended at a concentration of $1 \times 10^6$ $ml^{-1}$ in phenol red-free medium, and labelled with 5 nM 5-(SAENTA-$x_8$)-fluorescein at room temperature (RT) for 10 min in the dark, in the presence or absence of 2.5 μM NBMPR as previously described (Jamieson *et al.*, 1993). Fluorescence was measured using an Epics Elite flow cytometer (Coulter, Hialeah, FL, USA) fitted with a 488 nm argon laser, collecting emission with a bandpass filter centred at 525 nm. Simultaneous measurement of 5-(SAENTA-$x_8$)-fluorescein binding and cellular DNA content was done using an air-cooled helium–cadmium laser emitting at 325 nm to excite the vital DNA dye Hoechst-33342. Forward angle and right-angle scatter signals were used for live cell gating and a minimum of 10 000 viable cells were examined for each sample.

### Results

As previously reported (Jamieson *et al.*, 1993), 5-(SAENTA-$x_8$)-fluorescein bound to the membranes of viable cells from all four cell lines with high affinity, achieving saturation at a concentration of approximately 5 nM (data not shown). Coincubation with a 500-fold excess of the non-fluorescent compound NBMPR, a tight binding inhibitor of *es*, reduced cell fluorescence to that of background autofluorescence, confirming that 5-(SAENTA-$x_8$)-fluorescein is a specific, high-affinity ligand for *es*.

Dual staining with 5-(SAENTA-$x_8$)-fluorescein and the vital DNA dye Hoechst-33342 enabled the investigation of changes in *es* expression throughout the cell cycle. Because competition was observed between Hoechst-33342 uptake and 5-(SAENTA-$x_8$)-fluorescein binding, the concentration of Hoechst-33342 used was reduced from 10 to 1.0 μM, and the concentration of 5-(SAENTA-$x_8$)-fluorescein increased from 5 to 80 nM. Despite this relatively high 5-(SAENTA-$x_8$)-fluorescein concentration, binding was shown to be specific as demonstrated by NBMPR displacement. As shown in Figure 1, there was an increase in *es* expression during S-phase, which persisted into $G_2$ phase.

Modulation of *es* expression was investigated using antimetabolite drugs with differing mechanisms of action. Each drug was used at a concentration that was growth inhibitory while maintaining cell viability as assessed by the laser light scattering pattern observed by flow cytometry. For the cytotoxic agents 5-FU and ara-C this corresponded approximately to the $IC_{50}$ concentration as determined by clonogenic survival. The modulation of *es* expression on the surface of MGH-U1 cells by 5-FU, ara-C, TdR and APC following a 24 h exposure is shown in Figure 2. Ara-C, TdR and APC had no appreciable effect on *es* expression, whereas 5-FU and hydroxyurea increased the expression of *es*, by up to 10-fold with 40 μM 5-FU in MGH-U1 cells. The effects of hydroxyurea were further investigated using MGH-U1, CEM, Jurkat, and AML2 cells (Figure 3). In all of the cell

**Figure 1** (**a**) Cell cycle relationship of *es* expression in CEM human T-lymphoblasts, using dual labelling with 5-(SAENTA-$x_8$)-fluorescein and Hoechst-33342. (**b**) Specificity control using 500 × excess NBMPR.

lines, 24 h exposure to 50 and 200 μM hydroxyurea increased *es* expression. This effect was reversed by simultaneous treatment with the protein translation inhibitor cycloheximide (2.5 μg $ml^{-1}$), indicating that the effect of hydroxyurea is to increase *de novo* synthesis of *es*, rather than to cause its redistribution in membranes. Treatment of CEM cells with 2.5 μg $ml^{-1}$ cycloheximide alone resulted in a decrease in *es* expression to 29% of control value at 24 h, and 6% of control at 48 h, giving a half-life of approximately 12 h for *es* on the cellular membrane.

Exponentially growing cells incubated in either nucleoside-free or nucleoside-rich media for 48 h showed no difference in *es* expression or modulation. Thus, preformed nucleosides did not alter the expression of *es*, consistent with the lack of modulation of *es* expression observed with TdR in our studies.

### Discussion

Mammalian cells possess several specific transport elements that mediate both the entry and exit of nucleosides (Paterson *et al.*, 1981; Wohlhueter and Plagemann, 1982). Substrate

**Figure 2** Effects of anti-cancer agents on *es* expression in MGH-U1 cells. Drugs were exposed for 24 h at the concentrations indicated. Each point represents the mean of at least three separate experiments ± s.d.

**Figure 3** Effects of hydroxyurea on *es* expression exposed for 24 h in various cell lines. Each point represents the mean of at least three separate experiments ± s.d.

specificity for *es* is quite broad in that structurally diverse molecules, including purine and pyrimidine ribonucleosides and deoxyribonucleosides, synthetic nucleosides and nucleoside analogues, can be transported. The relevance of *es* to cancer treatment has two aspects: reduced expression might be associated with resistance to treatment using modified nucleosides that enter cells via *es*, such as ara-C (Wiley *et al.*, 1985) or 2-chloro-2′-deoxyadenosine, whereas the increased capacity to salvage preformed nucleosides from extracellular fluid is believed to confer resistance to other antimetabolite drugs, such as methotrexate (MTX) (Cabral *et al.*, 1984; Wadler *et al.*, 1987) and 5-FU (Grem and Fischer, 1989), that inhibit the *de novo* synthesis of DNA precursors. Attempts have been made to exploit nucleoside salvage pathways in order to improve chemotherapy results. For example, potentiating the uptake and phosphorylation of ara-C by depleting the endogenous dCTP pool with hydroxyurea (Walsh *et al.*, 1980; Rauscher and Cadman, 1983) or inhibiting these processes with agents such as dipyridamole to increase the effectiveness of MTX or 5-FU (Cabral *et al.*, 1984; Wadler *et al.*, 1987; Grem and Fischer, 1989).

In this paper we have confirmed that 5-(SAENTA-$x_8$)-fluorescein is a high-affinity, specific ligand for *es*. Because we postulated that surface expression of nucleoside transporter might increase during DNA synthesis we examined its correlation with cell cycle distribution using dual labelling with 5-(SAENTA-$x_8$)-fluorescein and the vital DNA dye Hoechst-33342. As shown in Figure 1, there was an approximately 2-fold increase in *es* during the cell cycle. Similar results have been reported in HeLa cells synchronised by mitotic detachment, in which the number of [$^3$H] NBMPR binding sites increased 2- to 3-fold as cells progressed from $G_1$ phase through S-phase of the cell cycle (Cass *et al.*, 1979). The comparatively small increase in *es* during the cell cycle argues against the expression being regulated to meet a demand for DNA precursors during S-phase. Using the protein synthesis inhibitor cycloheximide we obtained a half-life for *es* in the plasma membrane of 12 h. This agrees closely with the half-life of loss of nucleoside transport capacity observed during induced differentiation of HL60 cells (Chen *et al.*, 1986). If the synthesis of nucleoside transporter were indeed synchronised with the onset of S-phase, as we had originally postulated, this comparatively long half-life might explain why the increase in *es* expression during the cell cycle is only approximately 2-fold.

Expression of *es* can be modulated by treatment with growth-inhibitory concentrations of certain antimetabolites. Of particular interest is the increase in *es* expression following exposure to hydroxyurea, which inhibits ribonucleotide reductase and depletes deoxynucleotide triphosphate pools generally, and to 5-FU, which depletes dTTP. These increases were large compared to the natural increase in *es* expression during the cell cycle, and are therefore unlikely to be explained simply by a build up of cells in S-phase. Thymidine itself is an allosteric inhibitor of ribonucleotide reductase, and depletes dATP, dCTP and dGTP while increasing the dTTP pool, and treatment with this agent produced no increase in *es* expression. Aphidicolin and ara-C, which directly inhibit DNA synthesis, also had no significant effect on *es* expression.

We interpret these data as showing that cells can actively regulate the expression of *es* on the surface membrane, and that this regulation involves a feedback control which is sensitive to the supply of deoxynucleotides, particularly dTTP. It is well established that depletion of DNA precursors can increase the activity of salvage pathways, but this is usually considered to be due to an effect on the specific kinases which phosphorylate nucleosides, thereby trapping them inside the cell. Although it has been suggested that the efficiency of *es* is such that the initial rate of uptake is unlikely to be rate limiting for the salvage pathway, the large increases in *es* expression following exposure to 5-FU or hydroxyurea *in vitro* suggest that under some circumstances its capacity might be inadequate for maintenance of DNA synthesis. This up-regulation of *es* has important implications as a possible adaptive mechanism leading to antimetabolite drug resistance in cancer patients. Further studies should therefore be aimed at elucidating the mechanisms by which *es* expression is regulated *in vitro* and determining the relationship between the levels of expression and the response to chemotherapy in cancer patients. Because of the specificity and ease of use, flow cytometric measurement of fluorescent derivatives of SAENTA appears to offer a powerful new method for studying the role of *es* in response to antimetabolite drugs.

**Abbreviations**

NT, nucleoside transporter; *es*, equilibrative NBMPR-sensitive nucleoside transporter; NBMPR, *S*-(*p*-nitrobenzyl)-6-thioinosine; SAENTA, 5′-*S*-(2-aminoethyl)-$N^6$-(4-nitrobenzyl)-5′-thioadenosine; ara-C, cytosine arabinoside; ara-CTP, ara-C triphosphate; 5-FU, 5-fluorouracil; TdR, thymidine; APC, aphidicolin: dTTP, thymidine-5′-triphosphate; dTMP, thymidine-5′-monophosphate, thymidylate; dATP, 2-deoxyadenosine-5′-triphosphate; dCTP, 2-deoxycytidine-5′-triphosphate; dGTP, 2-deoxyguanosine-5′-triphosphate.

**Acknowledgements**

We wish to thank FW Tan for his assistance in handling the leukaemia cell lines, S Chow for her expertise in flow cytometry and ARP Paterson for many helpful discussions.

**References**

AGBANYO FR, VIJAYALAKSHMI D, CRAIK JD, GATI WP, MCADAM DP, ASAKURA J, ROBINS MJ, PATERSON ARP AND CASS CE. (1990). 5′-S-(2-Amino-ethyl)-$N^6$-(4-nitrobenzyl)-5′-thioadenosine (SAENTA), a novel ligand with high affinity for polypeptides associated with nucleoside transporter. *Biochem. J.* **270**, 605–614.

BELT JA. (1983). Heterogeneity of nucleoside transport in mammalian cells. Two types of transport activity in L1210 and other cultured neoplastic cells. *Mol. Pharm.* **24**, 79–484.

BELT JA, MARINA NM, PHELPS DA AND CRAWFORD CR. (1993). Nucleoside transport in normal and neoplastic cells. *Adv. Enzyme Regul.* **33**, 235–252.

CABRAL S, LEIS S, BOVER L, NEMBROT M AND MOSDOH J. (1984). Dipyrimidole inhibits reversion by thymidine of methotrexate effect and increases drug uptake in Sarcoma 180 cells. *Proc. Natl Acad. Sci. USA*, **81**, 3200–3203.

CASS CE, GAUDETTE LA AND PATERSON ARP. (1974). Mediated transport of nucleosides in human erythrocytes: specific binding of the inhibitor nitrobenzylthioinosine to nucleoside transport sites in the erythrocyte membrane. *Biochim. Biophys. Acta*, **345**, 1–10.

CASS CE, DAHLIG E, LAU EY, LYNCH TP AND PATERSON ARP. (1979). Fluctuations in nucleoside uptake and binding of the inhibitor of nucleoside transport, nitrobenzylthioinosine, during the replication cycle of HeLa cells. *Cancer Res.* **39**, 1245–1252.

CHEN SF, CLEAVELAND JS, HOLLMAN AB, WIEMANN MC, PARKS RE JR. AND STOECKLER JD. (1986). Changes in nucleoside transport of HL-60 human promyelocytic cells during N,N-dimethylformamide induced differentiation. *Cancer Res.* **46**, 3449–3455.

CRAWFORD CR AND BELT JA. (1991). Sodium-dependent, concentrative nucleoside transport in Walker 256 rat carcinosarcoma cells. *Biochem. Biophys. Res. Commun.*, **175**, 846–855.

CRAWFORD CR, NG CYC AND BELT JA. (1990). Nucleoside transport in L1210 murine leukemia cells. Evidence for three transporters. *J. Biol. Chem.*, **265**, 13730–13734.

ERLICHMAN C AND VIDGEN D. (1984). Cytotoxicity of adriamycin in MGH-U1 cells grown as monolayer cultures, spheroids, and xenographts in immune-deprived mice. *Cancer Res.*, **44**, 5369–5375.

GREM JL AND FISCHER PH. (1989). Enhancement of 5-fluorouracil's anticancer activity by dipyridamole. *Pharmacol. Ther.*, **40**, 349–371.

JAMIESON GP, BROCKLEBANK AM, SNOOK MB, SAWYER WH, BUOLAMWINI JK, PATERSON ARP AND WILEY JS. (1993). Flow cytometric quantitation of nucleoside transporter sites on human leukemia cells. *Cytometry*, **14**, 32–38.

NOTTEBROCK H AND THEN R. (1977). Thymidine concentrations in serum and urine of different animal species and man. *Biochem. Pharmacol.*, **26**, 2175–2179.

PATERSON ARP, KOLASSA N AND CASS CE. (1981). Transport of nucleoside drugs in animal cells. *Pharmacol. Ther.*, **12**, 515–536.

PLAGEMANN PGW, WOHLHUETER RM AND WOFFENDIN C. (1988). Nucleoside and nucleobase transport in animal cells. *Biochim. Biophys. Acta*, **947**, 405–433.

PRESSACCO J AND ERLICHMAN C. (1993). Combination studies with 3′-azido-3′-deoxythymidine (AZT) plus ICI D1694: cytotoxic and biochemical effects. *Biochem. Pharmacol.*, **46**, 1989–1997.

RAUSCHER F III AND CADMAN E. (1983). Biochemical and cytokinetic modulation of L1210 and HL-60 cells by hydroxyurea and effect on 1-β-D-arabinofuranosylcytosine metabolism and cytotoxicity. *Cancer Res.*, **43**, 2688–2693.

RODEN M, PATERSON ARP AND TURNHEIM K. (1991). Sodium-dependent nucleoside transport in rabbit intestinal epithelium. *Gastroenterology*, **100**, 1553–1562.

WADLER S, SUBAR M, GREEN MD, WIERNIK PH AND MUGGIA FM. (1987). Phase II trial of oral methotrexate and dipyridamole in colorectal carcinoma. *Cancer Treat. Rep.*, **71**, 821–824.

WALSH CT, CRAIG RW AND AGARWAL RP. (1980). Increased activation of 1-β-D-arabinofuranosylcytosine by hydroxyurea in L1210 cells. *Cancer Res*, **40**, 3286–3292.

WHITE JC, RATHMELL JP AND CAPIZZI RI. (1987). Membrane transport influences the rate of accumulation of cytosine arabinoside in human leukemia cells. *J. Clin. Invest.*, **79**, 380–387.

WILEY JS, JONES SP, SAWYER WH AND PATERSON ARP. (1983). Cytosine arabinoside transport by human leukaemic cells. *Eur. J. Clin. Oncol.*, **19**, 1067–1074.

WILEY JS, TAUPIN J, JAMIESON GP, SNOOK M, SAWYER WH AND FINCH LR. (1985). Cytosine arabinoside transport and metabolism in acute leukemias and T cell lymphoblastic lymphoma. *J. Clin. Invest.*, **75**, 632–642.

WOHLHUETER RM AND PLAGEMANN PGW. (1982). On the functional symmetry of nucleoside transport in mammalian cells. *Biochim. Biophys. Acta*, **689**, 249–260.